# cGAS-STING-TBK1 Signaling Promotes Valproic Acid-Responsive Human Cytomegalovirus Immediate-Early Transcription during Infection of Incompletely Differentiated Myeloid Cells

**DOI:** 10.3390/v16060877

**Published:** 2024-05-30

**Authors:** Emily R. Albright, Robert F. Kalejta

**Affiliations:** Institute for Molecular Virology and McArdle Laboratory for Cancer Research, University of Wisconsin—Madison, Madison, WI 53706, USA; rfkalejta@wisc.edu

**Keywords:** HCMV, herpes, cGAS-STING-TBK1, PRR, transcription, innate immunity, latency

## Abstract

Repression of human cytomegalovirus (HCMV) immediate-early (IE) gene expression is a key regulatory step in the establishment and maintenance of latent reservoirs. Viral IE transcription and protein accumulation can be elevated during latency by treatment with histone deacetylase inhibitors such as valproic acid (VPA), rendering infected cells visible to adaptive immune responses. However, the latency-associated viral protein UL138 inhibits the ability of VPA to enhance IE gene expression during infection of incompletely differentiated myeloid cells that support latency. UL138 also limits the accumulation of IFNβ transcripts by inhibiting the cGAS-STING-TBK1 DNA-sensing pathway. Here, we show that, in the absence of UL138, the cGAS-STING-TBK1 pathway promotes both IFNβ accumulation and VPA-responsive IE gene expression in incompletely differentiated myeloid cells. Inactivation of this pathway by either genetic or pharmacological inhibition phenocopied UL138 expression and reduced VPA-responsive IE transcript and protein accumulation. This work reveals a link between cytoplasmic pathogen sensing and epigenetic control of viral lytic phase transcription and suggests that manipulation of pattern recognition receptor signaling pathways could aid in the refinement of MIEP regulatory strategies to target latent viral reservoirs.

## 1. Introduction

Human cytomegalovirus (HCMV) is a widespread herpesvirus that causes severe disease in immunocompromised individuals and birth defects in neonates, and it has been associated with cardiovascular diseases and certain tumors [1,2]. HCMV establishes a lifelong infection in its hosts, in part through the establishment of latency in incompletely differentiated cells of the myeloid lineage, such as CD34+ hematopoietic progenitor cells and CD14+ monocytes [3,4,5]. Latent reservoirs are not cleared by either the adaptive immune response or existing antiviral therapies [6,7]. The ability of latently infected cells to persist despite the robust HCMV-specific T-cell response generated in most immunocompetent individuals [8,9,10] suggests that latently infected cells are likely not visible to adaptive immune surveillance.

The ability of HCMV to avoid immune detection during latency is achieved, in large part, by the suppression of viral lytic antigen expression. Indeed, a key tenet of the establishment and maintenance of latency is the repression of viral immediate-early (IE) gene expression, in part via silencing of the viral major immediate-early promoter and enhancer (MIEP) that controls transcription of the major viral transactivators IE1 and IE2 [11,12]. The viral IE1 protein is the major target of CD8+ T-cell responses [13] and drives productive replication [14,15] via the functions of over 200 viral proteins, many of which are also recognized by the adaptive immune system [13,16]. In contrast, during latent infection, the vast majority of lytic phase antigens are not detectable.

During de novo lytic infection in differentiated cells, the MIEP is rapidly activated by tegument-delivered viral transactivators and is associated with a transcriptionally activating chromatin structure [17,18]. In contrast, during the establishment of latency, the MIEP is assembled into heterochromatin and transcription from this locus is suppressed [11,17,19]. Both cellular and viral proteins contribute to the repression of the MIEP during latency [11,20]. The viral tegument transactivator pp71 remains endosome-associated in latently infected cells [21,22,23,24] and thus fails to reach the nucleus and degrade the cellular repressor Daxx, allowing for cellular defenses to install repressive heterochromatin at the MIEP [18]. Latency-associated viral gene products such as US28 and UL138 further enforce repression during latency by preventing the association of transcriptional activators [25,26,27,28,29,30] and promoting the recruitment of restrictive chromatin factors [31] to the MIEP.

Elucidating the mechanisms underlying how chromatin structures are written at the MIEP is important not only for understanding the molecular basis of latency but also for devising strategies for therapeutic intervention. We and others have shown that small molecule inhibitors of histone deacetylases (HDACs) such as valproic acid (VPA) relieve repression of the MIEP and allow for IE transcription and IE1 protein accumulation in undifferentiated myeloid cells where the MIEP would otherwise be silenced and latency established [23,24,27,32,33,34,35,36]. Indeed, small molecule epigenetic modifiers, including HDAC inhibitors, have emerged as potential therapeutics for targeting latent reservoirs using a “shock and kill” strategy [6,7,37]. An understanding of the mechanisms by which these small molecules regulate viral gene expression could help inform and refine such strategies.

We have previously shown that the latency-associated viral UL138 protein inhibits VPA-responsive transcription from the HCMV MIEP during the establishment of latency in incompletely differentiated myeloid cells, in part by preventing the recruitment of CtBP1, KDM1a and KDM6b to the MIEP and preserving transcriptionally repressive epigenetic histone methylations (H3K9me2/3 and H3K27me3) [27]. More recently, we showed that the MIEP must contain functional NFkB or CRE sites to be repressed by UL138 [35]. VPA-responsive IE gene expression in the absence of UL138 also requires functional NFkB or CRE sites [35], suggesting that an understanding of UL138 function could provide insights into the mechanisms controlling VPA-responsiveness.

In addition to its known role in repressing the MIEP, UL138 inhibits the cGAS-STING-TBK1 pathway and limits the accumulation of IFNB transcripts during both lytic and latent HCMV infection [38]. In mammalian cells, the cGAS-STING-TBK1 pathway is a pattern recognition receptor (PRR) pathway of the innate immune system that recognizes double-stranded DNA and initiates a type I interferon (IFN-I) response, primarily via activation of IRF3 [39,40,41]. Interestingly, the activation of cGAS-STING-TBK1 also leads to the activation of NFkB [42,43,44,45] and, in some contexts, of MAPK pathways that can act upstream of CREB [41,46], the same two pathways known to be important for the VPA-responsiveness of the MIEP [35]. Here, we show that the cGAS-STING-TBK1 pathway promotes VPA-responsive IE gene expression during HCMV infection of incompletely differentiated myeloid cells in the absence of UL138. Inactivation of cGAS-STING-TBK1 phenocopied UL138 expression by preventing NFkB recruitment to the MIEP and suppressing VPA-responsive IE gene expression. Taken together, our findings suggest that control of viral IE gene expression and the IFN-I response during the establishment of latency may be mechanistically linked.

## 2. Materials and Methods

### 2.1. Cells and Viruses

THP1 monocytes (ATCC TIB-202) were maintained between 2 × 10^5^ and 1 × 10^6^ cells/mL in RPMI-1640 (11875119; Life Technologies) supplemented with 10% fetal bovine serum (Sigma, St. Louis, MO, USA or GeminiBio, West Sacramento, CA, USA) and 1% PSG (G1146; Sigma, St. Louis, MO, USA) at 37 °C in a 5% CO_2_ atmosphere. The THP1-derived knockouts of cGAS and STING were gifts from Viet Hornung (LMU Munich) and have previously been described [47]. THP1-Dual (cat. no: thpd-nfis), THP1-Dual-TBK1 KO (cat. no: thpd-kotbk), and THP1-Dual-IKKe KO (cat. no: thpd-koikke) were purchased from Invivogen. Where indicated, PMA (P1585; Sigma, St. Louis, MO, USA) was added at a final concentration of 100 ng/mL to induce differentiation of THP1 cells into macrophage-like cells.

Viral stocks of HCMV AD169 and a derivative in which a C-terminally HA-tagged UL138 is expressed from its native putative promoter [27] were derived from BAC clones transfected into deidentified primary human fibroblasts (NHDFs, Clonetics, San Diego, CA, USA) and concentrated through a sorbitol cushion, as previously described [35,38]. All the virus stocks used in this study had been passaged 3 times or fewer. The THP1s were pre-treated with or without 1 mM VPA (Sigma, St. Louis, MO, USA) for 3 h prior to infection with HCMV at an MOI of 1 (as determined by plaque assay on NHDFs) in a minimal volume for 1 h at 37 °C before being returned to normal culture volumes in the presence or absence of 1 mM VPA for the indicated amount of time. Where indicated, the virus stocks were UV-inactivated prior to infection by exposure to a 245 nm light source at 0.12 J/cm^3^ on ice for 2 min in a Stratalinker 2400 (Stratagene, La Jolla, CA, USA). Where indicated, 10 μg/mL Ru521 (inh-ru521; Invivogen, San Diego, CA, USA), 10 μM BX795 (tlrl-bx7; Invivogen), or 1 μM diABZI (tlrl-diabzi-2; Invivogen, San Diego, CA, USA) were added after the first hour of infection and maintained throughout infection.

### 2.2. Cell Viability Assays

For the cell viability assays, THP1 cells were treated with 1 mM VPA, 10 μg/mL Ru521, 10 μM BX795, 1 μM diABZI, or an appropriate vehicle control for 24 h. Cell viability was determined using the Cell Titer Glo Assay (G7570; Promega, Madison, WI, USA), according to the manufacturer’s instructions. Luciferase activity was measured using a Turner Biosystems luminometer (Turner Biosystems, Sunnyvale, CA, USA) with a 0.5 s integration time and normalized to the control-treated samples. Media alone (no cells) served as a background control.

### 2.3. Western Blots

For Western blot analysis, equal numbers of cells were harvested, washed with PBS, and lysed with SDS lysis buffer (1%SDS, 2% b-mercaptoethanol). The samples were then boiled and equal amounts were run on SDS-PAGE gels and transferred to 0.2 μm nitrocellulose membranes (GE Healthcare, Chicago, IL, USA). The membranes were blocked with 5% BSA/TBST and probed with appropriate primary and secondary antibodies, as previously described [35], and imaged and quantitated with a LiCor Odyssey Fc with ImageStudio v.2.1.10 software. The mouse monoclonal antibodies against HCMV IE1 [48] and pp71 [49] have previously been described. The antibodies against pIRF3 Ser396 (clone 4D4G; 4947), cGAS (clone D1D3G; 15102), STING (clone D2P2F; 13647), TBK1 (3013), and IKKe (clone D20G4; 2905) were obtained from Cell Signaling Technologies (Danvers, MA, USA). The antibody against alpha-tubulin (clone DM1A; 05-829) was from Sigma and the antibody against total IRF3 (clone FL-425; sc-9082) was from Santa Cruz Biotechnology (Santa Cruz, CA, USA).

### 2.4. RNA Isolation and RT-qPCR

For the transcript analysis, cells were harvested and washed with PBS and the total RNA was isolated with an IBI RNA minikit (IB47323), following the manufacturer’s directions. Equal amounts of total RNA were treated with DNase and converted to cDNA using the Maxima H minus supermix with the dsDNase system (M1682; Life Technologies, Carlsbad, CA, USA), following the manufacturer’s directions. Equal amounts of cDNA were then used for the qPCR using the iTaq SYBR green universal supermix (1725125; Bio-Rad, Hercules, CA, USA) on an ABI 7900HT instrument with SDS2.4 software. Primers specific to HCMV IE (UL122/123 exon 3), human IFNB1, and GAPDH have previously been described [38]. Primers to amplify the differentiation specific transcripts GCSFR, ID2, and CD11B have also been described previously [50]. Transcript levels were normalized to GAPDH and calculated relative to the control sample using the ΔΔCt method, as previously described [35,38]. Standard curves were run with each plate to verify the amplification efficiency and that the samples were within the linear range of the assay. Melting curve analysis verified the presence of a single product with the expected melting temperature.

### 2.5. Chromatin Immunoprecipitation (ChIP) Assays

ChIP assays were performed as previously described [27,51]. Briefly, VPA-treated, infected cells were harvested, washed with PBS, and fixed with 1% formaldehyde, followed by quenching with 125 mM glycine. The nuclei were isolated and chromatin was sheared by sonication with a QSonica Q700 sonicator (QSonica, Newtown, CT, USA). Chromatin from approximately 1 million cells per reaction was incubated with 4 µg of ChIP-grade anti-NFkB p65/RelA antibody (17-10060; Millipore, Burlington, MA, USA) or matched IgG control. Chromatin/antibody complexes were collected using Protein A+G magnetic beads (16-663; Millipore, Burlington, MA, USA). The beads were subsequently washed, crosslinks reversed, and DNA isolated with a QIAQuick PCR cleanup kit (28104; Qiagen, Germantown, MD, USA). The input and immunoprecipitated DNA was quantitated by qPCR as described above using primers specific to the HCMV MIEP (5′CTT ATG GGA CTT TCC TAC TTG and 5′CGA TCT GAC GGT TCA CTA A) [27,51,52] or IFNB1 promoter region (5′ TGGCACAACAGGTAGTAGCGCACA and 5′TGGAGAAGCACAACAGGAGAGCA).

## 3. Results

### 3.1. VPA Treatment Activates an IFN-I Response during HCMV Infection of Incompletely Differentiated Myeloid Cells

**Figure 1:**
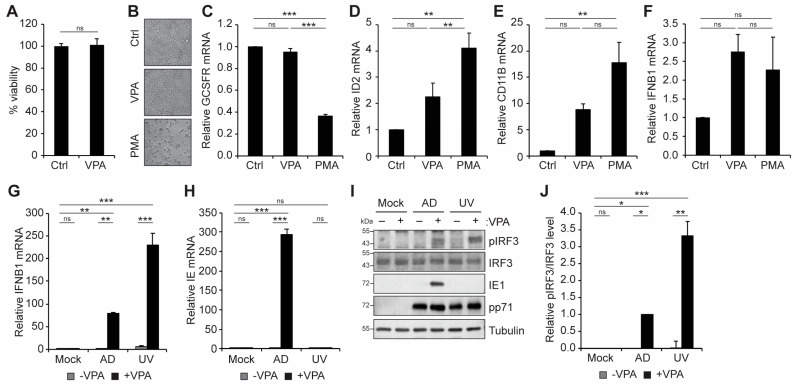
**VPA treatment activates an IFN-I response during HCMV infection of incompletely differentiated myeloid cells.** (**A**) Viability of THP1 cells untreated (Ctrl) or treated with 1 mM VPA for 24 h, plotted relative to untreated control cells (Ctrl). (**B**) Morphology of THP1 cells left untreated (Ctrl) or treated with 1 mM VPA or 100 ng/mL PMA for 24 h. (**C**–**F**) THP1 cells treated as in panel B and analyzed by RT-qPCR for GCSFR (**C**), ID2 (**D**), CD11B (**E**), or IFNB1 (**F**) transcripts. (**G**,**H**) THP1 cells pre-treated without (−) or with (+) 1 mM VPA were mock infected or infected with AD169 (AD) or UV-inactivated AD169 (UV) at an MOI of 1 for 18 h and analyzed by RT-qPCR for IFNB1 (**G**) or viral IE (**H**) transcripts. (**I**) THP1 cells treated and infected as in panel G were harvested at 18 h post infection and analyzed by Western blot for the indicated proteins. (**J**) Quantitation of pIRF3 protein levels from panel I, normalized to total IRF3 levels and plotted relative to VPA treated WT infected samples from the same blot. All bar graphs represent the mean ± SEM from three biological replicates. *: *p* < 0.05, **: *p* < 0.01, ***: *p* < 0.001, ns: *p* > 0.05 by one-way ANOVA with Tukey’s post hoc test for multiple comparisons.

The histone deacetylase inhibitor VPA is known to activate viral IE gene expression following HCMV infection of myeloid cells where latency would otherwise be established [24,27,32,33,35,53,54], most potently from viral strains lacking virally encoded inhibitors of the MIEP, such as UL138 [24,27,54]. Treatment of THP1 monocytes with VPA did not affect cell viability (Figure 1A), nor did it affect the morphology of THP1 cells (Figure 1B). In contrast, treatment with PMA, a known inducer of THP1 cell differentiation [55,56], resulted in the cells becoming adherent and displaying morphological changes (Figure 1B) consistent with differentiation. As expected, PMA treatment also resulted in changes in the transcript levels of the differentiation-associated genes [50] granulocyte colony-stimulating factor receptor (GCSFR; Figure 1C), inhibitor of DNA binding 2 (ID2; Figure 1D), and cluster of differentiation 11B (CD11B; Figure 1E), whereas treatment with VPA did not affect transcript levels of GSCFR (Figure 1C) or ID2 (Figure 1D) and had only modest effects on CD11B expression (Figure 1E). We conclude that treatment with VPA does not affect the viability or differentiation state of THP1 monocytes.

Inhibition of HDAC1 has previously been reported to trigger a cGAS-STING-dependent IFN-I response in both human and porcine epithelial cells that restricts pseudorabies virus infection [57]. Treatment of uninfected THP1 cells with either VPA or PMA did not significantly affect the basal level of IFNB1 transcripts (Figure 1F). However, following infection with HCMV, VPA treatment resulted in a dramatic increase in IFNB1 transcript levels (Figure 1G). Interestingly, this induction was observed following infection with both live and UV-inactivated virus, indicating that VPA-responsive IFNB1 transcript accumulation depends on viral infection, but not de novo viral gene expression (Figure 1G,H). Consistent with the activation of IFNB, we observed an increase in the levels of phosphorylated IRF3 with VPA treatment during infection with either live or UV-inactivated virus (Figure 1I,J). We conclude that VPA treatment enhances the IFN-I response during infection with HCMV.

### 3.2. cGAS and STING Promote VPA-Responsive IFN-I and Viral IE Gene Expression during HCMV Infection of Incompletely Differentiated Myeloid Cells

**Figure 2:**
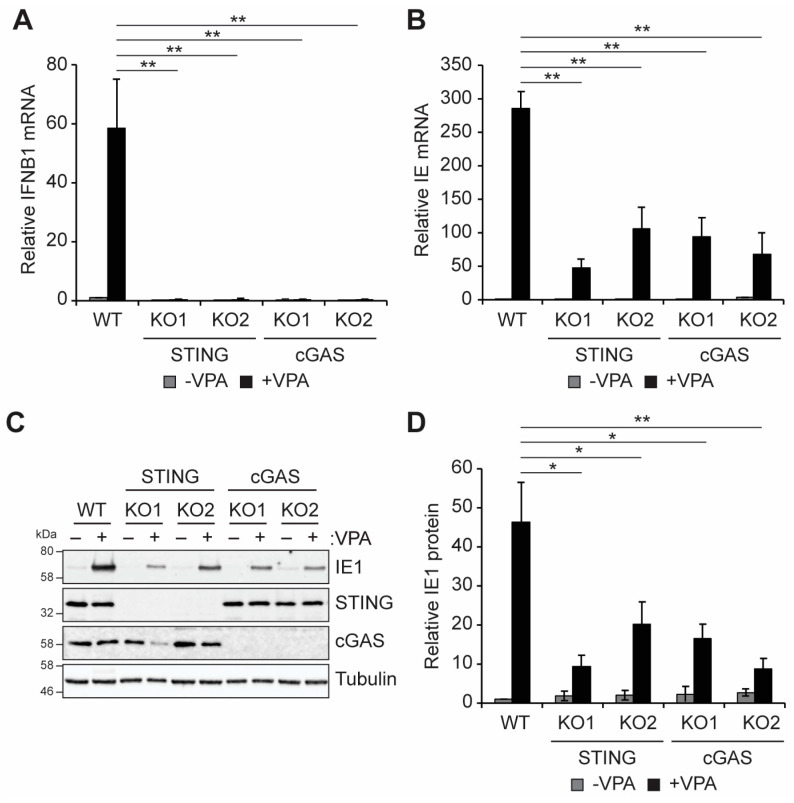
**Knockout of cGAS or STING impairs VPA-responsive IE gene expression during HCMV infection of incompletely differentiated myeloid cells**. (**A**,**B**) Wild-type (WT) THP1 cells or two independent clones of STING or cGAS knockout (KO) cells were pre-treated without (−) or with (+) 1 mM VPA and infected with AD169 at an MOI of 1 for 18 h and analyzed by RT-qPCR for IFNB1 (**A**) or viral IE (**B**) transcripts. n = 3 (**C**) WT and STING or cGAS knockout THP1s were treated and infected as in panel A and analyzed by Western blot with the indicated antibodies. n = 4. (**D**) Quantitation of IE1 protein levels from panel C, normalized to Tubulin levels and plotted relative to untreated WT infected samples from the same blot. n = 4. All bar graphs represent the mean ± SEM from the indicated number of biological replicates. *: *p* < 0.05, **: *p* < 0.01 by one-way ANOVA with Tukey’s post hoc test for multiple comparisons.

Induction of IFN-I responses in THP1 monocytes and THP1-derived macrophages infected with HCMV strain TB40/E was previously reported to be dependent on cGAS-STING signaling [58]. We confirmed that IFNB1 transcript accumulation following VPA treatment in undifferentiated THP1 monocytes infected with HCMV strain AD169, which lacks UL138, was also dependent on cGAS and STING (Figure 2A). THP1 monocytes with CRISPR-mediated knockout of either STING or cGAS [47] accumulated fewer IFNB1 transcripts compared to wild-type cells following infection with HCMV AD169 (Figure 2A). Interestingly, while wild-type cells supported the robust induction of viral IE transcripts in the presence of the HDAC inhibitor VPA, cells lacking either STING or cGAS had reduced VPA-responsive IE transcript accumulation (Figure 2B). IE1 protein accumulation also occurred in response to VPA treatment in wild-type THP1 cells but was diminished in the absence of either cGAS or STING (Figure 2C,D). We conclude that cGAS and STING promote both IFN-I and VPA-responsive viral IE gene expression during HCMV infection of incompletely differentiated myeloid cells.

**Figure 3:**
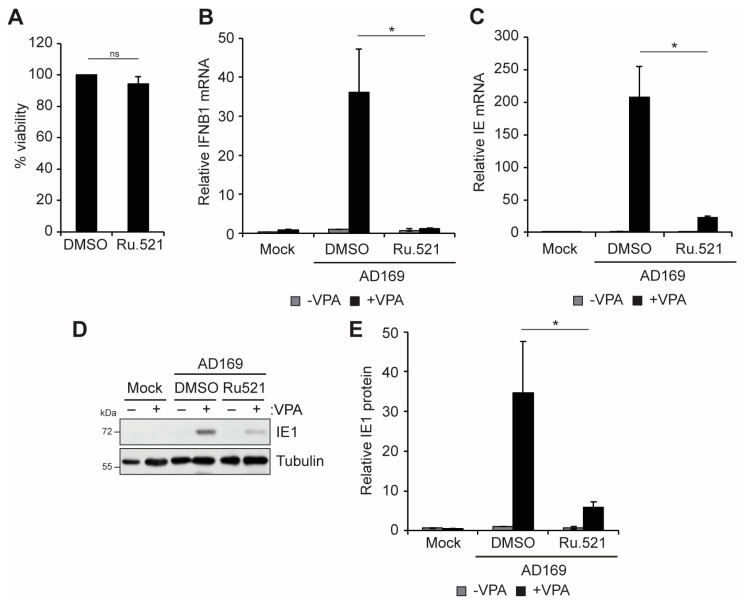
**cGAS activity promotes VPA-responsive IE gene expression during HCMV infection of incompletely differentiated myeloid cells**. (**A**) Viability of THP1 cells treated with DMSO or 10 μg/mL Ru.521 for 24 h, plotted relative to DMSO treated controls from the same experiment. n = 3. (**B**,**C**) THP1 cells were pre-treated without (−) or with (+) 1 mM VPA and either mock infected or infected with HCMV AD169 at an MOI of 1 in the presence of DMSO or 10 μg/mL Ru.521 for 18 h and analyzed by RT-qPCR for IFNB1 (**B**) or viral IE (**C**) transcripts. n = 3. (**D**) THP1 cells treated and infected as in panel A and analyzed by Western blot with the indicated antibodies n = 4. (**E**) Quantitation of IE1 protein levels from panel D, normalized to Tubulin levels and plotted relative to DMSO treated WT infected samples from the same blot. n = 4. All bar graphs represent the mean ± SEM from the indicated number of biological replicates. *: *p* < 0.05, ns: *p* > 0.05 by Student’s *t*-test.

To further validate the role of cGAS-STING signaling in the control of IFNB and viral IE gene expression during infection, we used a small molecule inhibitor of cGAS activity, Ru.521 [59,60]. Treatment with Ru.521 did not affect cell viability (Figure 3A), but it inhibited the induction of IFNB1 transcripts following infection with AD169 (Figure 3B), confirming that cGAS activity is required for the IFN-I response to HCMV infection in THP1 cells. Inhibition of cGAS also significantly reduced VPA-responsive IE transcript (Figure 3C) and IE1 protein (Figure 3D,E) accumulation. Taken together, these data suggest that the cGAS/STING activity promotes VPA-responsive IFNB1 and viral IE gene expression during HCMV infection of incompletely differentiated myeloid cells.

### 3.3. TBK1 Promotes IFN-I and VPA-Responsive Viral IE Gene Expression during HCMV Infection of Incompletely Differentiated Myeloid Cells

**Figure 4:**
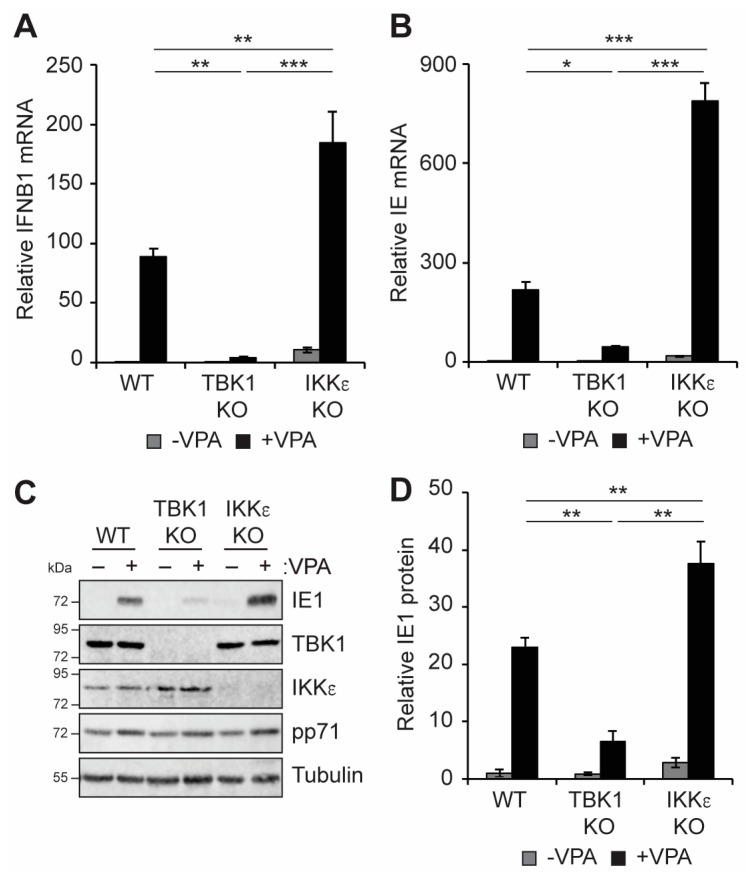
**Knockout of TBK1 inhibits VPA-responsive IE gene expression during HCMV infection of incompletely differentiated myeloid cells**. (**A**,**B**) Wild-type (WT), TBK1 knockout (TBK1 KO), or IKKe knockout (IKKe KO) THP1 cells were pre-treated without (−) or with (+) 1 mM VPA and infected with AD169 at an MOI of 1 for 18 h and analyzed by RT-qPCR for IFNB1 (**A**) or viral IE (**B**) transcripts. (**C**) WT and knockout THP1s were treated and infected as in panel A and analyzed by Western blot with the indicated antibodies. (**D**) Quantitation of IE1 protein levels from panel C, normalized to Tubulin levels and plotted relative to untreated WT infected samples from the same blot. All bar graphs represent the mean ± SEM from four biological replicates. *: *p* < 0.05, **: *p* < 0.01, ***: *p* < 0.001 by one-way ANOVA with Tukey’s post hoc test for multiple comparisons.

Canonical cGAS-STING pathway signaling results in the translocation of STING from the ER to the Golgi, where it activates TBK1 to induce the phosphorylation and activation of downstream IRF3 and NFkB, factors that promote IFNB1 transcription [39,40,41]. Knockout of TBK1, but not of the highly related kinase IKKε, inhibited the accumulation of IFNB1 transcripts in infected THP1 cells (Figure 4A). Similar to the loss of cGAS and STING (Figure 2), VPA-responsive IE transcript (Figure 4B) and IE1 protein (Figure 4C,D) accumulation was diminished in TBK1 knockout cells. We conclude that TBK1, like cGAS and STING, is required for IFNB accumulation and promotes VPA-responsive IE gene expression. In contrast, knockout of IKKε resulted in slightly enhanced IE transcript (Figure 4B) and IE1 protein (Figure 4C,D) accumulation in response to VPA treatment. Taken together, these data suggest that the role in promoting IFNB1 and VPA-responsive IE gene expression downstream of cGAS/STING during HCMV infection is specific to TBK1 and that IKKε may instead restrict viral gene expression during HCMV infection of incompletely differentiated myeloid cells.

**Figure 5:**
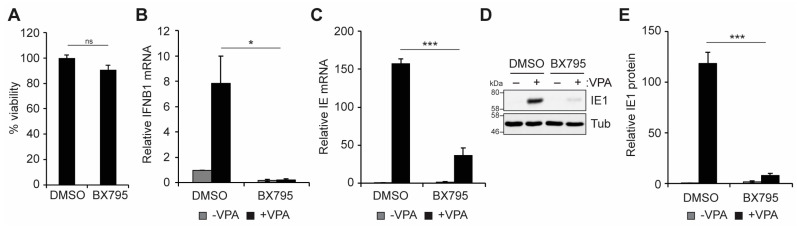
**TBK1/IKKe activity promotes VPA-responsive IE gene expression during HCMV infection of incompletely differentiated myeloid cells.** (**A**) Viability of THP1 cells treated with DMSO or 10 µM BX795 for 24 h, plotted relative to DMSO-treated controls from the same experiment. n = 3 (**B**,**C**) THP1 cells were pre-treated without (−) or with (+) 1 mM VPA and infected with AD169 at an MOI of 1 in the presence of DMSO or 10 µM BX795 for 18 h and analyzed by RT-qPCR for IFNB1 (**B**) or viral IE (**C**) transcripts. n = 4. (**D**) THP1 cells treated and infected as in panel A and analyzed by Western blot with the indicated antibodies. n = 3. (**E**) Quantitation of IE1 protein levels from panel D, normalized to Tubulin levels and plotted relative to DMSO treated WT infected samples from the same blot. n = 3. All bar graphs represent the mean ± SEM from the indicated number of biological replicates. *: *p* < 0.05, ***: *p* < 0.001, ns: *p* > 0.05 by Student’s *t*-test.

We further confirmed the role of TBK1 in regulating IFN-I and VPA-responsive IE gene expression using a small molecule inhibitor of TBK1/IKKε activity, BX795 [61]. Treatment with BX795 did not affect cell viability (Figure 5A), but it inhibited the accumulation of IFNB1 transcripts (Figure 5B) and VPA-responsive IE transcript (Figure 5C) and IE1 protein (Figure 5D,E) accumulation. We conclude that TBK1 activity is required for IFN-I and VPA-responsive IE gene expression during HCMV infection in THP1 monocytes.

### 3.4. Activation of STING Is Sufficient to Induce Viral IE Gene Expression in Incompletely Differentiated Myeloid Cells

**Figure 6:**
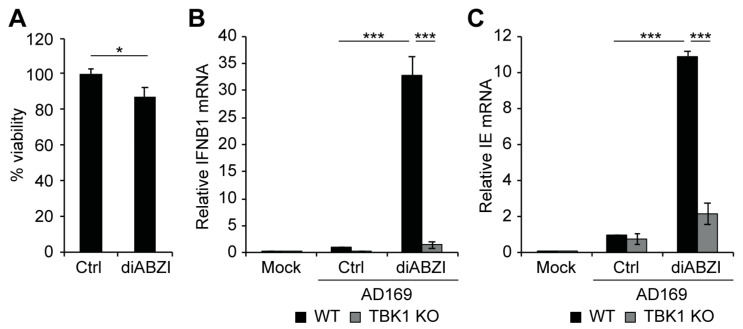
**Activation of STING is sufficient to induce viral IE gene expression in a TBK1-dependent manner.** (**A**) Viability of THP1 cells untreated (Ctrl) or treated with 1 µM diABZI for 24 h, plotted relative to untreated controls from the same experiment. (**B**,**C**) Wild-type (WT) or TBK1 knockout (TBK1 KO) THP1 cells were mock infected or infected with AD169 an MOI of 1 in the absence (Ctrl) or presence of 1 µM diABZI for 18 hrs and analyzed by RT-qPCR for IFNB1 (**B**) or viral IE (**C**) transcripts. All bar graphs represent the mean ± SEM from three biological replicates. *: *p* < 0.05, ***: *p* < 0.001 by one-way ANOVA with Tukey’s post hoc test for multiple comparisons.

Our data suggest that the cGAS/STING/TBK1 pathway promotes both an IFN-I response and viral IE gene expression following infection of THP1 monocytes, at least in the presence of VPA. To test the hypothesis that activation of this pathway is sufficient to drive viral IE gene expression, we treated cells with the small molecule STING agonist diABZI [62]. Treatment with diABZI did not substantially affect cell viability (Figure 6A), but it resulted in a robust induction of both IFNB1 (Figure 6B) and viral IE transcripts (Figure 6C). As expected, treatment with diABZI did not induce either IFNB1 or viral IE transcripts in the absence of TBK1 (Figure 6B,C). We conclude that pharmacological activation of STING is sufficient to activate both IFN-I and viral IE gene expression in a TBK1-dependent manner.

### 3.5. Loss of cGAS or Expression of UL138 Impairs Recruitment of NFkB to the Viral MIEP and IFNB1 Promoter

**Figure 7:**
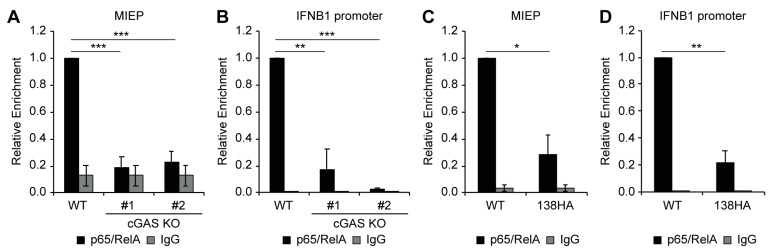
**Loss of cGAS or expression of UL138 impairs recruitment of NFkB to the viral MIEP and IFNB1 promoter.** (**A**,**B**) ChIP assays for NFkB p65/RelA (black bars) or matched IgG control (gray bars) at the MIEP (**A**) or IFNB1 promoter (**B**) in WT THP1 or two independent clones of cGAS knockout (KO) cells pretreated with 1 mM VPA and infected with wild-type AD169 at an MOI of 1 for 18 hpi. Enrichment relative to WT control from the same experiment is shown. (**C**,**D**) ChIP assays for NFkB p65/RelA (black bars) or matched IgG control (gray bars) at the MIEP (**C**) or IFNB1 promoter (**D**) in WT THP1 cells pretreated with VPA and infected with either wild-type AD169 (WT) or AD169 expressing HA-tagged UL138 (138HA). Enrichment relative to WT infection control from the same experiment is shown. All bar graphs represent the mean ± SEM from three biological replicates. *: *p* ≤ 0.05, **: *p* < 0.01, ***: *p* < 0.001 by one-way ANOVA with Tukey’s post hoc test for multiple comparisons.

Canonical cGAS-STING-TBK1 signaling culminates in the activation of transcription factors IRF3 and NFkB, which then migrate to the nucleus to activate expression of IFN-I genes, including IFNB1 [39,40,41]. The HCMV MIEP is not known or predicted to encode binding sites for IRF3, but it does encode four binding sites for NFkB [12,63], which we have previously shown to be important for VPA-responsive IE gene expression in myeloid cells [35]. Thus, we hypothesized the cGAS-STING-TBK1 signaling promotes viral IE gene expression by promoting NFkB activation and recruitment to the MIEP. To test this hypothesis, we measured the binding of the NFkB subunit p65/RelA to the MIEP in wild-type or cGAS KO THP1 cells by chromatin immunoprecipitation (ChIP) assay. NFkB was recruited to the MIEP in VPA-treated wild-type cells but not in two independent clones of cGAS KO THP1 cells (Figure 7A), indicating that cGAS promotes NFkB recruitment to the MIEP. In agreement with the observation that IFNB1 transcript accumulation is dependent on cGAS-STING-TBK1 (Figure 2A, Figure 3B, Figure 4A and Figure 5B), NFkB binding to the IFNB1 promoter was also abolished in cGAS knockout THP1 cells (Figure 7B).

**Figure 8:**
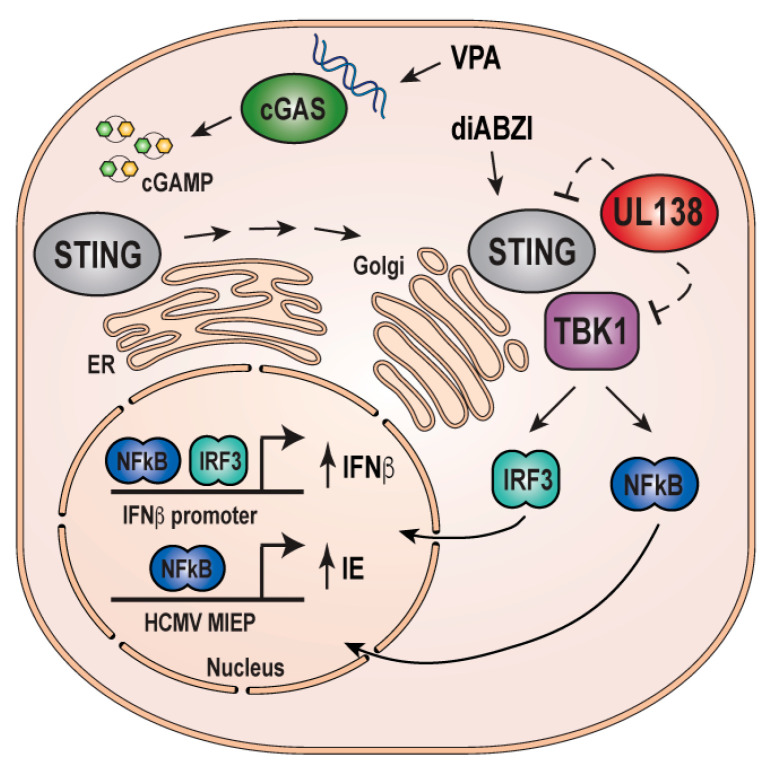
**Model for VPA-responsive IFN-I and viral IE gene expression mediated by cGAS-STING-TBK1.** Activation of the cGAS-STING-TBK1 pathway in HCMV-infected cells drives the transcription of cellular IFNB1 and viral IE1/2. In the presence of UL138, the pathway is inhibited, reducing the accumulation of IFNB1 and IE1/2. Model adapted from [38].

We have previously shown that UL138 inhibits VPA-responsive IE gene expression [27,53,54] and inhibits the cGAS-STING-TBK1 pathway, resulting in a diminished IFN-I response [38]. Because our data suggest that inhibition of the cGAS/STING/TBK1 pathway in the absence of UL138 impairs VPA-responsive IE gene expression (Figure 2, Figure 3, Figure 4 and Figure 5), we hypothesized that expression of UL138 would phenocopy inhibition of cGAS-STING-TBK1 and prevent recruitment of NFkB to both the MIEP and the IFNB1 promoter. As expected, NFkB was bound to both the MIEP (Figure 7C) and the IFNB1 promoter (Figure 7D) in VPA-treated THP1 cells infected with wild-type AD169. However, NFkB binding to both the MIEP (Figure 7C) and the IFNB1 promoter (Figure 7D) was reduced upon infection of an AD169-derivative that re-expresses UL138 [27]. We conclude that UL138 expression phenocopies inhibition of cGAS-STING-TBK1 to inhibit the activation of both IFN-I and VPA-responsive IE gene expression during the establishment of latency (Figure 8).

## 4. Discussion

Repression of viral IE gene expression is a key characteristic of the establishment and maintenance of HCMV latency [11,20,64,65], and manipulation of viral IE gene expression with small molecules is a promising approach to targeting latent reservoirs [6,7,32,33]. Our observation here that cGAS-STING-TBK1 signaling promotes VPA-responsive IE gene expression during the establishment of HCMV latency suggests that PRR detection and signaling could protect against latent infection by both inducing an IFN-I response and promoting the expression of viral antigens that can render infected cells visible to adaptive responses. Even transient expression of viral IE genes can lead to recognition and killing by existing HCMV-specific T-cells present in seropositive individuals [32,33]. Thus, cGAS-STING-TBK1-mediated induction of viral IE gene expression may not only prevent the establishment of latency but also render infected cells susceptible to T-cell-mediated clearance.

Our observation that the cGAS-STING-TBK1 pathway is responsible for the accumulation of IFNB1 transcripts in THP1 cells is consistent with previous work showing that this pathway is activated in myeloid cells infected with HCMV [58]. However, what triggers the activation of this pathway in latently infected cells or in response to VPA treatment remains to be determined. Previous work suggested that inhibition of HDAC1 could trigger a cellular DNA damage response that resulted in the activation of cGAS [57]; however, our data suggest that, at least in THP1 cells, viral infection is required for VPA to induce an IFN-I response (Figure 1G,I,J). Although the icosahedral capsid houses the viral genome within virions and during transport to the nucleus, degradation of some capsids may result in the leakage of viral genomes into the cytosol and allow for the sensing of viral DNA [66]. Alternatively, infection-induced sensing of cellular DNAs such as mitochondrial DNA that have leaked into the cytoplasm, which has been observed for other viruses [67,68,69,70], could also contribute to the activation of cytoplasmic cGAS in HCMV infected cells.

Though classically thought of as a sensor of cytoplasmic DNA [71], more recent studies have found cGAS in the nucleus of mammalian cells [72,73], where it appears to play a role in regulating the DNA damage response [73,74,75] and contribute to the sensing of both DNA and RNA virus infection [76,77]. Thus, it is possible that nuclear HCMV genomes, which are initially histone-free, could also serve directly or indirectly as a ligand for cGAS activation during infection.

While our data suggest that cGAS itself contributes to the IFN-I response and VPA-responsive IE gene expression (Figure 2 and Figure 3), the enzymatic product of cGAS activity, cGAMP, has been found to be incorporated into viral particles, including CMV particles [78], and thus direct delivery of cGAMP to latently infected cells could also contribute to the activation of STING/TBK1. Interestingly, in addition to interacting with STING and TBK1 [38], UL138 also downregulates MRP1 [79,80] which was recently shown to be a cGAMP exporter [81]. Whether the expression of UL138 alters the incorporation of cGAMP into viral particles or stocks remains to be determined.

Another nuclear DNA sensor, IFI16 [82,83,84], has been implicated in the detection of nuclear herpesviral genomes [85,86,87,88,89] and can signal downstream through STING in both cGAS-dependent [90,91] and cGAS-independent [92,93] pathways. Of note, IFI16 was also shown to promote activation of the HCMV MIEP in latently infected THP1 cells via recruitment of NFkB [28]. Interestingly, IFI16 is antagonized during the establishment of latency by viral US28-mediated downregulation [28]. US28, like UL138, represses the MIEP in incompletely differentiated myeloid cells and promotes latency [94], further supporting the idea that inhibition of PRR signaling may be an important mechanism for ensuring repression of viral IE genes during the establishment of latency. Whether IFI16 and cGAS act in the same or distinct signaling pathways in HCMV latently infected cells is not known.

In addition to UL138 and US28, the viral long non-coding RNA β2.7 also inhibits the MIEP during latency by preventing the induction of reactive oxygen species (ROS), which otherwise leads to the activation of NFkB and premature reactivation in infected cells [95,96]. Intriguingly, in some contexts, ROS accumulation can lead to the activation of cGAS-STING-TBK1 signaling [97]. Whether ROS accumulation contributes to the activation of cGAS-STING-TBK1 in HCMV-infected myeloid cells and whether viral RNA β2.7 also modulates this pathway remains to be determined.

TBK1 and IKKε are highly related members of the IKK family that activate both IRFs and NFkB [98,99]. Due to the high degree of homology and shared substrates, distinguishing between the roles of TBK1 and IKKε has generally proven challenging [99], although some distinctions have been observed [99,100]. Indeed, TBK1 and IKKε were reported to function redundantly to mediate STING-induced NFkB activation in mouse myeloid cells [43]. Our data, however, suggest that TBK1 and IKKε are not redundant for either IFNβ induction or VPA-responsive viral IE gene expression, at least not in the context of HCMV infection in THP1 cells (Figure 4). In fact, our results suggest that TBK1 and IKKε may have opposing effects in this context, as TBK1 promotes VPA-responsive IE gene expression and IFNB1 transcript accumulation while IKKε may instead repress it. Thus, our observations suggest that latent infection with HCMV could be a useful tool to probe the distinct functions of these highly related kinases. Additional work is needed to delineate the downstream effects of each of these kinases in the context of HCMV latency.

Although IFN-Is, including IFNβ, themselves restrict HCMV lytic replication [101,102,103] and thus might be thought to promote latency, our work here and that of others [28] suggests that PRR signaling upstream of IFN-I production can in fact enhance viral IE gene expression in myeloid cells via activation of the MIEP. Intriguingly, while UL138 expression reduced IFNB1 and CXCL10 accumulation downstream of cGAS-STING-TBK1 [38], it has also been reported to enhance the expression of some interferon stimulated genes (ISGs) at late times during productive infection [104], presumably by promoting the activation of STAT1 [105]. Interestingly, the activation of STAT1 by JAKs occurs downstream of exposure to IFN-Is [106,107,108]. Thus, UL138 may function to exquisitely fine-tune the innate immune response during latency to usurp the repressive effects of ISGs downstream of activated STAT1 while actively avoiding the upstream PRR signaling that would activate the MIEP.

The importance of the cGAS-STING-TBK1 pathway in controlling latency may extend to other latent viruses as well. Indeed, the combination of small molecule activators of cGAS-STING and histone deacetylase inhibitors triggered the reactivation of HIV-1 and the apoptosis of infected cells in vitro [109]. Whether the activation of cGAS-STING-TBK1 can similarly enhance the reactivation of HCMV, either via small molecule activators or inhibition of UL138 function, remains to be explored, although our data suggest that pharmacological activation of STING is at least sufficient to modulate viral IE gene expression (Figure 6). Furthermore, the ability of cGAS-STING-TBK1 signaling to activate both viral IE gene expression and a cellular IFN response could make manipulation of this pathway an attractive candidate for enhancing “shock and kill” strategies. Induction of IE1 protein expression could render infected cells susceptible to T-cell-mediated killing [32,33], while the concomitant production of IFN-Is and inflammatory signals may serve to bolster the immune response.

## Data Availability

All relevant data supporting the findings of this study are included in the article. Further inquiries can be directed to the corresponding author.
